# Early Smartphone App-Based Remote Diagnosis of Silent Atrial Fibrillation and Ventricular Fibrillation in a Patient with Cardiac Resynchronization Therapy Defibrillator

**DOI:** 10.3390/jcdd10010030

**Published:** 2023-01-14

**Authors:** Dagmar Kowal, Agnieszka Katarzyńska-Szymańska, Marek Prech, Błażej Rubiś, Przemysław Mitkowski

**Affiliations:** 1 Department of Clinical Chemistry and Molecular Diagnostics, Poznan University of Medical Sciences, 60-806 Poznan, Poland; 2 Doctoral School, Poznan University of Medical Sciences, 60-812 Poznan, Poland; 3 1st Department of Cardiology, Poznan University of Medical Sciences, 60-355 Poznan, Poland; 4 Department of Cardiology, Provincial Hospital, 64-100 Leszno, Poland

**Keywords:** Bluetooth, cardiac resynchronization therapy, heart failure, remote monitoring, silent atrial fibrillation, smartphone app, ventricular fibrillation

## Abstract

Due to distressing statistics concerning cardiovascular diseases, remote monitoring of cardiac implantable electronic devices (CIED) has received a priority recommendation in daily patient care. However, most bedside systems available so far are not optimal due to limited patient adherence. We report that smartphone app technology communicating with CIED improved the patient’s engagement and adherence, as well as the accuracy of atrial and ventricular arrhythmias diagnosis, thus offering more efficient treatment and, consequently, better patient clinical outcomes. Our findings are in concordance with previously published results for implantable loop recorders and pacemakers, and provide new insight for heart failure patients with an implanted cardiac resynchronization therapy defibrillator.

## 1. Introduction

In 2015, international physician societies recommended remote monitoring (RM) of cardiac implantable electronic devices (CIED) as a class I indication, supplementary to in-hospital-only device follow up [1]. The breakthrough was achieved due to a large body of evidence revealing that RM based on bedside transmitters reduced time from the onset of ventricular and supraventricular arrhythmias (including silent atrial fibrillation (AF)) to their evaluation [2]. The RM system provides scheduled follow ups and alarm-triggered transmissions, which are automatically sent to the manufacturer server, where they can be immediately analyzed by a medical team. The swift access to data and its evaluation by a healthcare specialist, followed by clinical intervention, could lead to reduced hospitalization time, reduced inappropriate diagnosis or therapies incidence rate and lowered workload. Consequently, it could lead to improved patient prognosis and clinical outcome [3,4]. However, the RM usage assessment (bedside systems) revealed that patient compliance has been suboptimal, ranging from 53% to 79% [5,6], which may lead to less effective and delayed treatment and, consequently, to heart failure (HF) progression. The main reason for the lower level of adherence was transmitter type, geographic localization, socioeconomic status, clinic facilities, and patient age and sex [7,8]. As suggested, these challenges might be addressed by the implementation of smartphone app technology, communicating with implantable loop recorders (ILRs) and pacemakers (PMs) via low energy Bluetooth (BLE) protocol. Consequently, application of the system caused excellent patient compliance and engagement and a reduction in traditional bedside transmitters’ limitations. That, in turn, led to the higher success rate of completed transmissions (94.6–92.0% for smartphone app transmissions vs. 56.3–87.1% for manual or wireless console transmissions) [9,10]. The smartphone app system was also more efficient in swift, appropriate diagnosis and effective data transmission. However, further evaluation, especially for HF patients with implanted cardiac resynchronization therapy defibrillator (CRT-D), is needed.

## 2. Detailed Case Description

**Figure 1:**
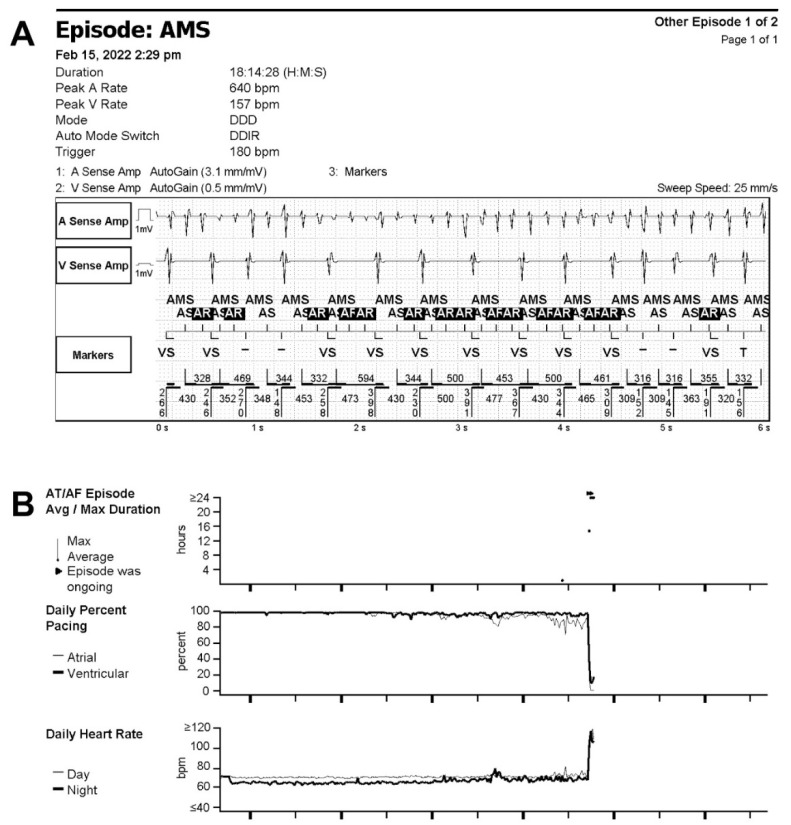
Remote diagnostics of atrial fibrillation via smartphone app: (**A**) Intracardiac electrogram of silent AF episode recorded by cardiac resynchronization therapy defibrillator and sent via Bluetooth smartphone app remote monitoring (RM) on 16 February 2022; (**B**) Diagnostics trends sent via RM presenting correlation between onset of AT/AF episode and significant drop in resynchronization therapy (Daily Percent Pacing) and increase in daily heart rate. AMS, auto mode switch.

We report the case of a patient with two episodes of different arrhythmias, silent AF and ventricular fibrillation (VF), detected via an implanted CRT-D device (Neutrino NxT HF, Abbott, Plymouth, MN, USA), which was directly transferred via BLE smartphone app (MyMerlinPulse, Abbott) to the RM network (Merlin.net, Abbott). A 67-yeard-old man with dilated cardiomyopathy, left bundle branch block (LBBB), left ventricular ejection fraction (LVEF) estimated on echocardiography at 25%, and chronic anticoagulation treatment after pulmonary veins isolation (PVI) was implanted with CRT-D and enrolled to RM on 10 June 2021. The patient was a casual smartphone user. An explanation of how RM works and support with app installation and pairing procedure with CIED was provided before discharge. The patient attended in-hospital follow ups every 6 months combined with remote follow ups scheduled every 2 months, including alarm transmission activated by arrhythmia onset. On 13 February 2022, the first alarm transmission triggered by the onset of atrial tachycardia (AT) was delivered. On 16 February 2022, another alarm transmission appeared, presenting an AF (Figure 1A) with no symptoms related to the onset of AF. On the same day, the patient was subjected to a remote interview. Diagnostics available in RM showed a significant decrease in resynchronization therapy (drop in biventricular pacing (BiV) therapy from 98% to 20%) and an increase in daily heart rate to 100–120 bpm (Figure 1B). The AT/AF burden reached 63% during this period.

**Figure 2:**
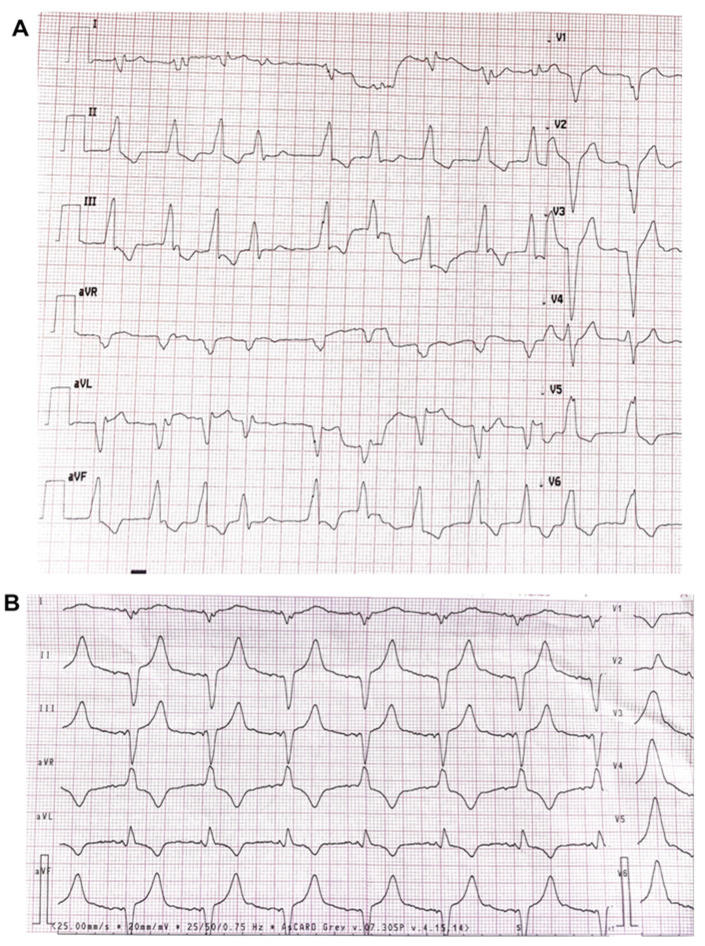
Patient ECG: (**A**) At admission to hospital on 3 March 2022. AF with average ventricular rate 112 bpm, LBBB, QRS width 170 ms. (**B**) At discharge from hospital on 5 March 2022. Atrial and biventricular pacing at 65 bpm.

**Figure 3:**
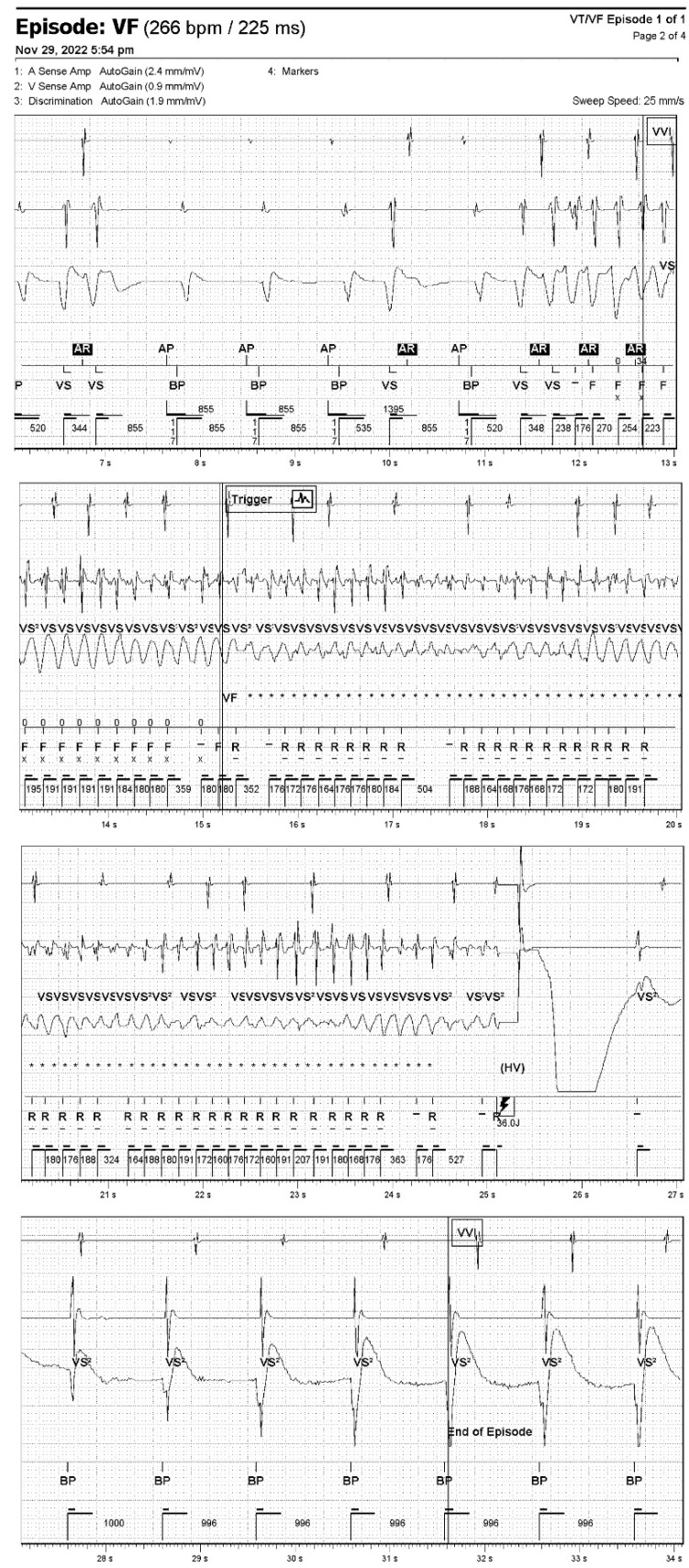
Remote diagnostics of ventricular fibrillation (VF) via smartphone app. Intracardiac electrogram of VF episode recorded by cardiac resynchronization therapy defibrillator and sent via Bluetooth smartphone app remote monitoring on 29 November 2022. *, capacitor charging.

The patient was admitted to the hospital on 3 March 2022. The hospital worked under a coronavirus disease 2019 (COVID-19) regimen, therefore earlier admission was not possible, but the patient had been on chronic anticoagulation treatment. Pharmacological treatment was altered and electrical cardioversion to restore sinus rhythm was performed (Figure 2). No further alarm transmissions were received until 22 July 2022, when the patient arrived for in-clinic follow up (12 months after implantation). The CRT-D interrogation did not reveal any new episodes of AT/AF, either. The echocardiographic examination showed a significant improvement in LVEF (40%). The Minnesota Heart Failure Questionnaire (baseline vs. 12 m) confirmed further progress in exercise tolerance and quality of life (QoL). The patient completed the questionnaire exploring his app-based RM experience, where he emphasized his satisfaction with direct involvement enabled by a user-friendly app. The second onset of AF was diagnosed via RM and was followed by elective hospitalization to perform cardioversion on 24 November 2022. On 29 November 2022, the patient contacted us via text message after brief loss of consciousness and possible shock delivery; he immediately forwarded the manual transmission of a potential tachyarrhythmia episode via the app. The swift access to data allowed the clinician to verify and confirm an appropriate diagnosis and high voltage therapy delivery for VF in less than an hour from its onset (Figure 3). The patient was admitted to the hospital the same day; acute coronary syndrome as well as thyroid and electrolyte imbalance were excluded. We decided not to perform a coronary angiogram as the one carried out in 2021 revealed no significant changes. We decided to add amiodarone to the treatment. After short hospitalization, the patient was released. Since that time (as of 23 December 2022), we have not observed any arrhythmic events in that patient.

## 3. Discussion

It is the first case, to the best of our knowledge, describing the implementation of a smartphone app-based RM in CRT-D device that led to the early identification of silent AF onset with a significant drop in BiV therapy, followed by hospital admission to restore sinus rhythm before the patient manifested hemodynamic decompensation and progressed to HF. It appears that the implementation of this technology might be beneficial for a broad range of life-threatening diseases and a broad group of patients. Studies conducted on a group of patients with implanted PMs and cardioverter-defibrillators (ICDs), without a previous history of stroke or AF, showed a 30–34.7% risk of de novo AF in a two-and-a-half-year follow-up [11,12]. This group of patients, who is at risk of thromboembolic complications or HF decompensation will benefit from RM development. The RM of CIED enables a reduction in the time from the onset of arrhythmias (ventricular tachycardia, VF, AF) to their evaluation, including episodes of clinically silent AF, and shows particular benefits for the prognosis. The COMPAS trial reported that RM was associated with a reduction in hospitalizations due to atrial arrhythmias (6 in the RM group vs. 18 in the control group) and thromboembolic complications and ischemic stroke (2 in the RM group vs. 8 in the control group) [13]. Although the patient described in our case report is after PVI and on chronic anticoagulation treatment that reduces the risk of a stroke, the applied smartphone app-base RM proved to be the right tool for fast and accurate diagnosis of silent AF. It corresponds with new guidelines for patients with AF from 2020 that oblige to anticoagulant treatment in the case of intracardiac recording (IEGM) of subclinical AF in patients with risk factors [14]. Similar results were also reported in a large randomized CONNECT study indicating an 18% reduction in hospitalization time in the group of patients subjected to remote monitoring, which corresponded with an estimated reduction in treatment costs of USD 1793 [15]. Improvement in fast and accurate notification of cardiac events provided by smartphone app-based RM can contribute to further treatment cost reduction. Our case strongly suggests that this could be an additional benefit of the RM approach, apart from the fact that patients show significantly higher QoL. We confirm that fast diagnosis and properly adjusted therapy significantly contribute to an increased recovery rate.

There is also another critical aspect. The smartphone app allowed the patient to view their transmission history: scheduled, alarm triggered and manual. It automatically reminded the patient to keep the app active and connected via BLE with CIED. The patient could verify the CIED model name and battery status. Importantly, the patient was able to contact us right after a VF episode before sending a manual transmission, because the app contains emergency contact information. Both transmission history and battery status were used most often by our patient, which shows his involvement, awareness and increased interest in health and motivation. We did not notice overuse of the RM system by excessive use of the manual transmission feature or contact info by the patient, but this may be an individual issue. Notably, the involvement of both parties led to medical team–patient partnership, which was perceived by both sides as a positive aspect. Our findings strongly correlate with the ALTITUDE study results, which was performed with the use of bedside transmitters. Authors showed that RM not only significantly reduced the time from onset to diagnosis/intervention (by 26–94%), but also reduced the number of hospital visits (by 12–68%). It also enhanced patients’ motivation and engagement and provided a stronger patient–physician collaboration [16].

### 3.1. RM and Mortality

Mortality (cardiovascular or all-cause) and hospitalization risk reduction was confirmed in numerous studies performed on large groups of patients remotely monitored (bedside systems) [17,18], although not all researchers corroborated the correlation between RM and all-cause mortality reduction in a group of HF patients [19,20]. However, the IN-TIME study revealed a dramatic reduction in HF patient mortality after RM implementation, with an increased frequency of transmission. Authors of that multi-center, randomized trial of 664 patients (1:1 RM arm/standard arm, 12-month observation) suggested that daily automated remote control of patients with implanted CIED and bedside transmitters significantly improved clinical outcome, showing a 69% reduction in the Packerscore endpoint—combined endpoint (mortality, hospitalization for HF, the New York Heart Association (NYHA) class). A total of 80% of the patients had good, continuous, uninterrupted quality of remote transmissions, which led to a conclusion that broad access to well-functioning RM should be provided in everyday clinical practice [2]. However, other studies evaluating the efficacy of RM based on bedside transmitters connected to the system via a mobile phone adapter or land line phone reported that even 45% of alerts was not transmitted to the medical team, mainly due to the faulty set up of the bedside transmitter. If the alerts were received, the time from the alert to medical team decision making was 4.6 days on average and only 84% of these alerts were transmitted within an acceptable time limit [15]. Similarly, inconsistency in transmissions was reported while patients were away for three or more consecutive days, leaving their bedside transmitter at home [2] We think that these obstacles can be overcome with current technology advancements: broad access to mobile data transfer and the availability of affordable and easy to operate smartphone technology. The smartphone app-based RM described in our case offered excellent adherence and compliance. Daily alert and trend checks triggering automated transmission, combined with vast accessibility, portability and universality of smartphone as a transmitter, provides an increased degree of uninterrupted quality of RM vs. bedside transmitters. Another aspect is patient education before discharge. This part is crucial for a patient’s understanding the benefits of RM and results in better adherence, as reported in the EVOLVO trial. It was shown that using bedside transmitters with ICDs significantly reduced the median time from the alert to review by the medical team to only 1.4 days [21].

### 3.2. Summary and Future Perspectives

Awareness of mobile technology is growing. People have become more familiar with apps monitoring daily health and sport activities. On a global scale, smartphone and smart wearables are becoming a fast-growing trend, especially due to access to low-cost mobile data transfers. The combination of novel app technology remotely monitoring CIED with numerous clinical data provides significant benefits for RM, as well as opportunities for creating new strategies and further advancing the effective management of HF patients.

The large quantity of remotely transferred data via mobile technology to the clinic shall be followed by improved organization, procedures and protocols allowing for a swift and efficient analysis. Another issue is building a medical team that will perform effective filtration and selection of patients that require an immediate call for an additional visit due to sudden cardiac arrhythmias or device dysfunctions [22]. Perhaps the next challenge is the development and implementation of artificial intelligence (AI) to improve the management and processing of diagnostic data collected in medical centers every day. Using AI could not only provide more efficient selection and sorting of incoming data, but also enable more detailed and broader association studies of more data, including novel biomarkers with unknown potential [23].

Notably, the COVID-19 pandemic revealed the importance of RM in many areas. The European Society of Cardiology (ESC) has introduced guidelines recommending the widespread use of telemonitoring to reduce the risk of infection in patients suffering from HF. It was recommended that patients with implemented remote control should postpone in-hospital visits, and remote control should be made available to patients without RM. Patients without symptoms and alarms could have their appointment rescheduled until the pandemic is over. Patients with electrode or battery dysfunction detected remotely via alarm transmissions (without the possibility of solving the problem remotely) were invited for an elective visit [24]. The American guidelines presented by the Heart Rhythm Society (HRS) went a step further, seeking to limit contact with medical staff through the extensive implementation of RM. It is recommended to strive for the implementation of remote control by implementing RM already at the time of CEID implantation, including all patients with CEID for remote monitoring at home, and continuing remote monitoring already in place and requesting full remote control before visiting the clinic. Only patients with potentially serious device or electrode dysfunction and the absolute need to reprogram the device should be directed to in-hospital visit. In addition, the use of other telemedicine tools to help assess clinical parameters (e.g., body weight, single-channel ECG, edema) should be strongly considered [25]. However, new challenges require novel solutions and it seems that broad access to mobile devices and BLE-based monitoring apps may provide an efficient and cost-effective solution. Our case presenting user-friendly smartphone app-based RM could provide important input, enhancing this strategy.

## 4. Conclusions

We show a single case report demonstrating that the implementation of new technologies based on smartphones, manageable apps and BLE communication may significantly improve the efficacy of RM. The user-friendly app (transmission history, clinic contact and manual transmission features) led to better compliance, patient engagement, and the high success rate of completed transmissions. Our findings are in concordance with previously published results for ILRs and PMs, and provide new insight for HF patients with implanted CRT-Ds. Early detection of arrhythmias, accurate diagnosis and reduced time to intervention and treatment led to improved patient clinical outcome, delayed heart disease progression and QoL enhancement. Thus, it seems that BLE-based RM may constitute one of the most important areas in telemedicine.

## Data Availability

Not applicable.
